# Centromere Repeats: Hidden Gems of the Genome

**DOI:** 10.3390/genes10030223

**Published:** 2019-03-16

**Authors:** Gabrielle Hartley, Rachel J. O’Neill

**Affiliations:** 1 Department of Molecular and Cell Biology, University of Connecticut, Storrs, CT 06269, USA; gabrielle.hartley@uconn.edu; 2 Department of Molecular and Cell Biology and Institute for Systems Genomics, University of Connecticut, Storrs, CT 06269, USA

**Keywords:** satellite, transposable element, repetitive DNA, chromosome evolution, centromere drive, genetic conflict, CENP-A, centromeric transcription

## Abstract

Satellite DNAs are now regarded as powerful and active contributors to genomic and chromosomal evolution. Paired with mobile transposable elements, these repetitive sequences provide a dynamic mechanism through which novel karyotypic modifications and chromosomal rearrangements may occur. In this review, we discuss the regulatory activity of satellite DNA and their neighboring transposable elements in a chromosomal context with a particular emphasis on the integral role of both in centromere function. In addition, we discuss the varied mechanisms by which centromeric repeats have endured evolutionary processes, producing a novel, species-specific centromeric landscape despite sharing a ubiquitously conserved function. Finally, we highlight the role these repetitive elements play in the establishment and functionality of de novo centromeres and chromosomal breakpoints that underpin karyotypic variation. By emphasizing these unique activities of satellite DNAs and transposable elements, we hope to disparage the conventional exemplification of repetitive DNA in the historically-associated context of ‘junk’.

## 1. Introduction

Specific types of repetitive segments within eukaryotic genomes are now recognized as critical to maintaining subspecialized genomic functions. Common elements within repetitive segments include both transposable elements (TEs) and satellite DNA [1], collectively representing a large portion of eukaryotic genomes [2,3]. Unlike TEs that are capable of moving within a genome and thus are often found dispersed (albeit not randomly; reviewed in [4]), satellite DNA consists of short stationary DNA sequences that tandemly repeat to form a larger array, often restricted to specific sub-regions of chromosomes [1,5]. Ranging from just a few base pairs to several megabases in length, satellite repetitive units comprise up to 10% of the human genome [6]; across eukaryotes, variation in copy number and satellite family diversity contributes to differences in total satellite DNA content among taxa, often with dramatic total satellite content differentials [5]. Despite the high degree of variation among species in both sequence diversity and overall content, satellite DNAs are collectively found most highly concentrated in the centromeric and pericentromeric regions of chromosomes [7]. While the exact functions of satellite DNA have not been fully realized, this incommensurate distribution of satellite DNA within the genome highlights the importance of satellite DNA in chromosome inheritance through participation in centromere function.

First described in the context of DNA content in eukaryotes by Kit et al. [8] and Seuoka et al. [9] in 1961, satellite DNA was discovered via ultracentrifugation of genomic DNA—Note: the first use of the term satellite as a genetic descriptor is attributed to Sergius Navashin in his 1912 study of secondary constrictions on the chromosomes of a hyacinth [10]. Following the centrifugation of DNA from animal tissue extracts across a cesium chloride density gradient, Kit et al. [8] described a satellite band that was clearly differentiated from the major band of genomic DNA. Due to the repetitive nature of the DNA within this band, this fraction displayed an observable shift in density and led to the first description of satellite DNA. Despite this traditional descriptor, the phrase satellite DNA has been used more broadly to describe all tandemly arranged repetitive DNA sequences [11] irrespective of their resolution on density gradients. Since their discovery, a number of different methods have been used to characterize tandem repeats, including C_0_T analysis, in which the rate of re-association of complementary DNA strands is used to identify the frequency of repetitive elements [12], and separation following restriction endonuclease treatment, in which digested genomic DNA is separated via electrophoresis on an agarose gel [13]. Modern molecular techniques including next-generation sequencing (NGS) and fluorescence in situ hybridization (FISH) have provided additional clarity in the identification and physical characterization of satellite DNA sequences. This review includes emerging discoveries about satellite array characteristics and the other types of repeats found within, model systems proven useful for studying the role of satellite DNA in genome evolution, and the intimate relationship between satellite DNA and TEs. In addition, this review examines the paradoxical link between divergent satellite DNA and conserved centromere function as well as the connection between repeats and the emergence of new centromeres during chromosome evolution.

## 2. A Brief Primer on Satellite DNA in a Chromosomal Context

While the term satellite DNA encompasses all tandem nucleotide repeats, this large category can be further divided into a number of different subcategories and families. In addition to larger tandem repeats, one such grouping of smaller repeats can be created based on the number of nucleotides existing in the core repetitive segment. Microsatellites, for example [14], consist of repeating units less than 10 nucleotides in length and constitute up to 3% of the human genome [15]. Minisatellites, often referred to as variable number tandem repeats (VNTRs) [16], consist of a 10 to 100 nucleotide unit repeating up to several hundred times. With several thousand minisatellite loci distributed throughout the human genome [17], minisatellites are found at a high frequency in telomeric regions [18]. Telomeres are also enriched for a specific microsatellite, (TTAGGG)_n_, which constitutes the bulk of telomeric sequences, extending for 9–15 kb on human chromosomes [19,20]. Nucleoproteins (TRF1, TRF2, and POT1) bind to these telomeric satellites to form the shelterin complex [21], which interacts with the ribonucleoprotein telomerase that contains the enzyme component telomerase reverse transcriptase (TERT) [22], and an RNA (TERRA) [23]. The resulting ‘cap’ distinguishes chromosome ends from DNA breaks requiring repair and thus protects the chromosome from end-degradation and interchromosomal fusions [24].

**Figure 1:**
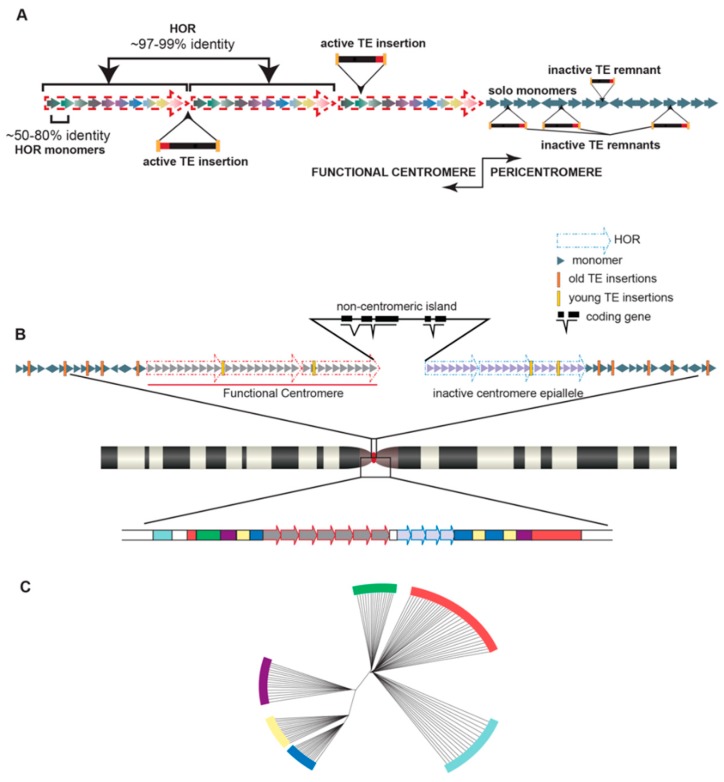
Overview of satellite DNA structure in a human centromere/pericentromere. (**a**) α satellite monomers (colored solid arrows) are organized into a repeating unit, called a higher order repeat (HOR) (red dashed arrows). In this example, 10 monomers are in each HOR (10-mers). HOR units are repeated in a chromosome-specific manner 100–1000 s of times within a functional centromere core. Within a single HOR, monomers share anywhere from 50–80% sequence identity with one another. The same monomer within different HORs in the same array may share up to 99% identity. Solo monomers (solid arrows) are found in the pericentromeric region and are highly variable in terms of sequence and orientation. Within the centromere, transposable elements (TE) insertions typically include recently active or active (hot) elements, while the TE insertions found in the pericentromere are older, inactive elements. (**b**) The core centromere structure (red dot, chromosome schematic) of human chromosomes (a generic chromosome ideogram is indicated, middle) consists of different α satellite arrays arranged in HORs (dashed arrows). Each HOR array may contain a different monomer number; in this example, the functional centromere (i.e., assembles CENP-A nucleosomes) at a 10-mer HOR (red dashed arrows). A 7-mer HOR is found nearby but is an inactive epiallele. Both HORs are separated by non-centromeric DNA, which may contain genes. α satellites are also found throughout the pericentromere (bottom schematic, different colored blocks). (**c**) Representative cladogram of the phylogenetic relationship of the non-HOR α monomers shown in (b). In this example, strata of newer satellites are closer to the HOR arrays, while older satellites are found more distally. Relative age of satellites is indicated by tree branch length; shorter branches are younger elements and deeper branches are older.

Perhaps the most notable satellite families in the human genome are those located at both pericentromeric and centromeric regions: α satellites. α satellites, found ubiquitously at all human centromeres, are a ~171 base pair unit, known as a monomer, with sequences that are 50–80% identical among all monomers within an array (repeated monomers in tandem) [25]. The core of the centromere, where the kinetochore will form and mediate microtubule attachment and faithful chromosome segregation, is functionally defined by the assembly of centromeric nucleosomes containing the centromere-specific histone 3, CENP-A [26]. In humans, this core is enriched for α satellite DNA [25]. While found as solo repetitive units scattered among other satellites in the pericentromeric regions of human chromosomes without higher organization, α satellites within human centromeres are tandemly repeated to form a block of satellites, called a higher order repeat (HOR). HORs are comprised of a set number of monomers that varies from 2 to 34 monomers ([25,27,28,29,30] and reviewed in [31]) in a largely chromosome-specific arrangement (Figure 1a). For example, the α satellite HOR blocks on chromosome 1 consist of 2 monomers [32], referred to as 2-mers, the HOR blocks on chromosome 7 consist of 6-mers [33], and the HOR blocks on the Y chromosome are 34-mers [34]. These HOR blocks are further repeated to form HOR arrays than can span megabases. Because of the highly repetitive nature of these centromeric HOR arrays with identities among HOR blocks nearing 99% in some cases [35] and high HOR copy numbers [36], centromeric regions have historically been refractive to characterization, at least in the context of genome assemblies [6]. 

Despite challenges associated with characterizing highly repetitive stretches of DNA, groups are uncovering variation in satellite DNA, both within the human reference genome and among different individuals, and identifying the functional consequences of these variants. α satellite monomers are classified into 12 consensus monomers (J1, J2, D1, D2, W1, W2, W3, W4, W5, M1, R1, and R2) [29,30,37,38,39], which are further grouped into five suprachromosomal families (SF1-5) [29,30,40]. A specific strata of satellites within each human chromosome was revealed by fine-scale mapping and sequence annotation of monomers and HORs [30,40], wherein highly homogenized and recently derived monomers are organized into HORs within the functional core of the centromere and the older, divergent monomers are organized further from the core and into the pericentromere (Figure 1b). In other words, the closer a satellite stratum is to the functionally defined core of the centromere, the younger and more homogenized the monomers within those HORs will be. It has thus been proposed that the α satellite strata are a phylogenetic record of the evolution of human centromeres, with the younger and more homogenized monomers closer to the functionally defined core of the centromere and older centromere remnants orbiting the central core, indicating the location and/or abandoned sequence of long-dead centromeres shared with our primate ancestors [40] (Figure 1c). 

**Figure 2:**
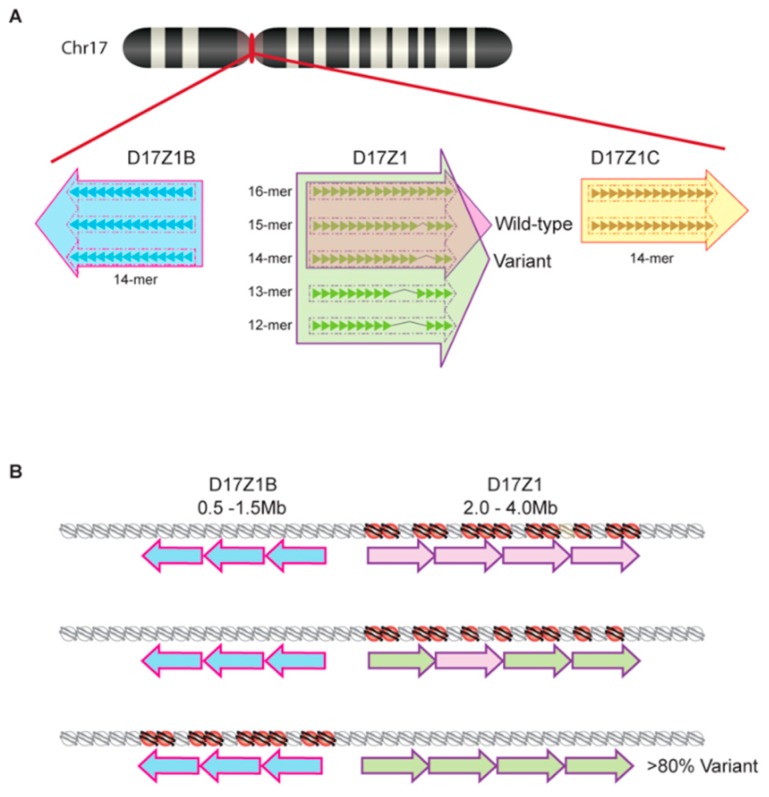
Chromosome 17 epialleles. (**a**) Ideogram of chromosome 17 (top). Zoom inset of epialleles showing monomer number for HORs and orientation. D17Z1B HORs carry 14 monomers, as do D17Z1C HORs. D17Z1 HORs are variable in the human population, with wild type epialleles containing 16-mer, 15-mer, and 14-mer HORs (pink) and variant epialleles containing wild type HORs in addition to 13-mer and 12-mer HORs (green). (**b**) Variation of the D17Z1 epiallele is linked to centromere activity. When the variation in D17Z1 increases, CENP-A nucleosomes (red) decrease; when variation exceeds 80%, the centromere assembles on the D17Z1B epiallele.

Once a satellite variant becomes dominant in a species, there is subsequent intrachromosomal homogenization that further distinguishes chromosome-specific arrays. Recent work in humans has also revealed that there is variation of chromosome-specific α satellite arrays among different individuals in the human population [41,42]. Aldrup-MacDonald et al. [41] describe variation within the α satellite DNA arrays of human chromosome 17 first characterized by several groups over the past few decades [40,43,44,45]. At this chromosome, the centromeric region contains three unique α satellite arrays arranged adjacently: D17Z1, D17Z1-B, and D17Z1-C (Figure 2). Among these three arrays, only one acts as the functional centromere and recruits CENP-A histones; thus, multiple, potentially functional arrays on one chromosome are known as epialleles [46]. In roughly 70% of individuals, the centromere is assembled at the 16-mer D17Z1 locus, while the remaining 30% of individuals display differential centromere assembly at the D17Z1 locus of one homolog and the 14-mer D17Z1-B locus of the other [46] (Figure 2a). While the D17Z1-B epiallele can support centromere assembly in human artificial chromosomes, no individual homozygous for this allele has yet been identified. Because of this, it is purported that those homozygous for the D17Z1-B epiallele represent a rare, yet functionally viable, variant in the human population [41]. Similarly, Miga et al. [42] have identified size and sequence satellite array variants on human chromosomes X and Y via their utilization of whole-genome shotgun sequencing in efforts to create centromeric reference models [47]. This ongoing work continues to build upon the foundational understanding of satellite array variation that has been characterized by others [48,49] and suggests that centromeric HOR variants are not a phenomenon exclusive to human chromosome 17.

While the underlying molecular foundation for the formation of centromeric epialleles remains unknown, Aldrup-MacDonald et al. [41] propose, based on their work with somatic cell hybrid lines, that genomic variation of satellite DNA is an influential factor dictating which epiallele will assemble centromeric nucleosomes [46]. Using restriction enzyme digestion and Southern blotting to identify variation in D17Z1, D17Z1-B, and D17Z1-C epialleles, Aldrup-MacDonald et al. [41] determined that larger D17Z1 satellite arrays were more likely to be both homogenous (wild type for the canonical 16-mer HOR) and the active site of centromere assembly. By using cytogenetic techniques like fluorescence in situ hybridization (FISH) and monitoring chromosome stability, it was determined that centromeres assembling at a highly variant D17Z1 array locus (containing a number of different HOR variants) were unstable while those assembling at the D17Z1-B locus remained stable despite D17Z1 variability [41] (Figure 2b). Furthermore, it was determined that these unstable centromeric locations had about half of the amount of centromeric proteins CENP-A and CENP-C present in comparison to stable centromeres [41]. These studies suggest that variant chromosome 17 epialleles do not perform equally and thus highlight the important role variation of satellite DNA might play in the maintenance of proper chromosome segregation.

## 3. Centromere Repeats Endure Unique Evolutionary Processes

**Figure 3:**
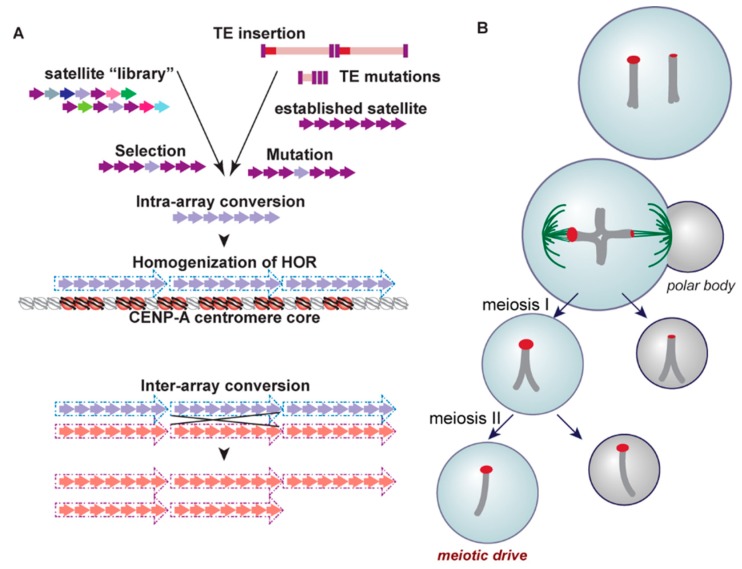
Schematic of the evolutionary mechanisms that impact centromere repeats. (**a**) Two models for the derivation of species-specific satellites are shown: (left) A satellite array evolves from a library of satellites, culminating in a dominant satellite; (right) TE insertion(s) followed by mutations, such as deletions, lead to the evolution of new satellites. In both cases, a homogenized array evolves through molecular drive mechanisms, such as intra-array concerted evolution. Stabilization of the arrays into HOR arrays defines the active centromere core, where CENP-A nucleosomes (red) are assembled. Other events, such as inter-array conversion, can lead to the spread of new HORs or changes in HOR copy number (bottom). (**b**) Two homologous chromosomes share the same satellite repeat (red), but one homolog experiences an expansion of that repeat through de novo mutations. During female meiosis, the larger centromere attracts more microtubules, resulting in the loss of the homolog with the weaker centromere into the polar body during meiosis I. The larger centromere is preferentially driven to the viable egg following unequal distribution of chromatids during meiosis II.

Although the presence of satellite DNA in centromeres is a shared characteristic found among many eukaryotic taxonomic groups, as is the protein cascade required for faithful chromosome segregation mediated by CENP-A, the underlying sequence of this satellite DNA is highly variable and largely species-specific [50,51,52,53,54,55]. Tandemly arrayed satellites within a single chromosome experience high rates of sequence turnover via concerted evolution, a non-independent process of molecular drive [56] (Figure 3a). Several mechanisms have been invoked to explain this observation, including nonhomologous and/or unequal crossing over [57], replication slippage [58], gene conversion [59], and rolling circle amplification and subsequent reinsertion ([60], reviewed in [61]). Such mechanisms impact sequence homogenization across an array as well as variation in overall array length. 

While tandemly arrayed sequences are not capable of transposition, a family of arrays appears to spread from one chromosome to another, rendering the centromere repeats of non-homologous chromosomes within a karyotype highly similar and phylogenetically closely related. For example, several pairs of human chromosomes share the same satellite arrays: chromosomes 1, 5, and 19 [62,63], 13 and 21 [64], and 14 and 22 [65]. Interestingly, chromosomes 13 and 21 in the chimpanzee share the same satellite array as is observed on the homologous chromosomes 13 and 21 in humans, but the 13/21 arrays of these two species are not orthologous [65,66], indicating some chromosomes efficiently evoke inter-chromosomal recombination in independent lineages [28]. How this occurs or why this appears restricted to a subset of chromosomes is not known. 

Homogenization of arrays is not linked specifically to the presence of tandem repeats. In fact, the only stratum of satellites across the centromere/pericentromere that experience forces of homogenization across an array, and thus carry HORs and high identity repeat units, is that of the recently derived and functional core [40] (Figure 3a). In other words, only the satellites that serve as the foundation for the kinetochore undergo continual homogenization, linking the assembly of the kinetochore to the homogenization process [37], and consequently, rapid evolution. It has been proposed that proteins facilitating homogenization, known as a kinetochore-associated recombination machine (KARM), have become integrated into the kinetochore complex, fostering this core-satellite specific homogenization process [28,40]. One candidate for this machine is topoisomerase II [40], a DNA decatenating enzyme that resides in the kinetochore during mitosis and initiates homologous recombination following the induction of DNA breaks [67]. 

What is the source material for new satellite arrays that seed within older arrays, eventually pushing them to outer, non-homogenized and highly variable strata? The library hypothesis [68] provides one explanation for how satellite DNA content at the centromere may diverge rapidly among closely related species (Figure 3a). In this scenario, extant but distinct centromeric repeats, representing a satellite library for a species, may independently expand or contract in copy number in different evolutionary lineages (be they chromosomes or species within a complex). If a repeat from this library finds itself in the core of the centromere, associated homogenization and expansion could result in the establishment of what appears to be a new satellite array [69,70,71,72,73,74]. In some cases, the seeding of a centromere from such a library is facilitated by chromosome rearrangement [75,76,77].

Another mechanism proposed to give rise to the variability of satellite sequences in different species is a meiotic drive model, known as centromere drive [78] (Figure 3b). As predicted by this model, satellite arrays attract more microtubules during female meiosis if the arrays experience accretion [79,80]. Preferentially sorted into the egg, these expanded satellite sequences are predicted to promote increased rates of evolution of centromere proteins, particularly CENP-A, which directly interacts with satellite DNA, through genetic conflict. Eventually, these divergent centromere proteins become highly prevalent in the population as they evolve to restore parity in meiosis [52,81]. In fact, this model is supported not only by the rapid evolution and variability of satellites in a variety of species, but by the positive selection of nucleic-acid interacting centromere proteins like CENP-A and CENP-C in plants, primates, and others [82,83,84,85,86]. This model is further supported by evidence that Robertsonian fusions with a single centromere are preferentially segregated due to a higher recruitment of CENP-A, Ncd80, and microtubules than their unfused mates [87]. Heterozygosity for these fusions has been observed to reduce male fertility, creating a selective pressure for the fixation of a new karyotype via a fitness cost ([88], reviewed in [89]). 

Despite the ability of the centromere drive model to explain the high variation observed in satellite sequence from one species to the next, this model does not offer a complete mechanism by which chromosomal evolution and karyotypic changes may occur, particularly when considering the circumstances of de novo centromere formation. Described in human patients presenting with an abnormal karyotype (reviewed in [90]), a neocentromere forms on an ectopic site on a chromosome when the original centromere is lost or inactivated, or the entire karyotype is unstable, as in cancer (e.g., [91]) —Note: It has been argued that the term neocentromere is incorrectly used to describe de novo centromeres that are kinetochore-competent [92]. The original use of the term neocentromere is attributed to describe subtelomeric heterochromatin blocks that behave similarly to centromeres but do not build a traditional kinetochore [93]. While stable neocentromeres can be fully functional in kinetochore assembly and thus maintain proper chromosome segregation, most lack the typical satellite DNA characteristic of centromeric regions [94,95,96,97,98,99]. Not only are functional neocentromeres devoid of satellite DNA, but in some cases, the original inactive centromere retains satellite arrays yet they no longer recruit centromere proteins (and thus are rendered non-functional) (reviewed in [90,100]). The identification of functional neocentromeres lacking satellite DNA spawned the prediction that satellite DNA is neither sufficient nor required for centromere function [101], despite its apparent ubiquity across taxonomic groups. 

**Figure 4:**
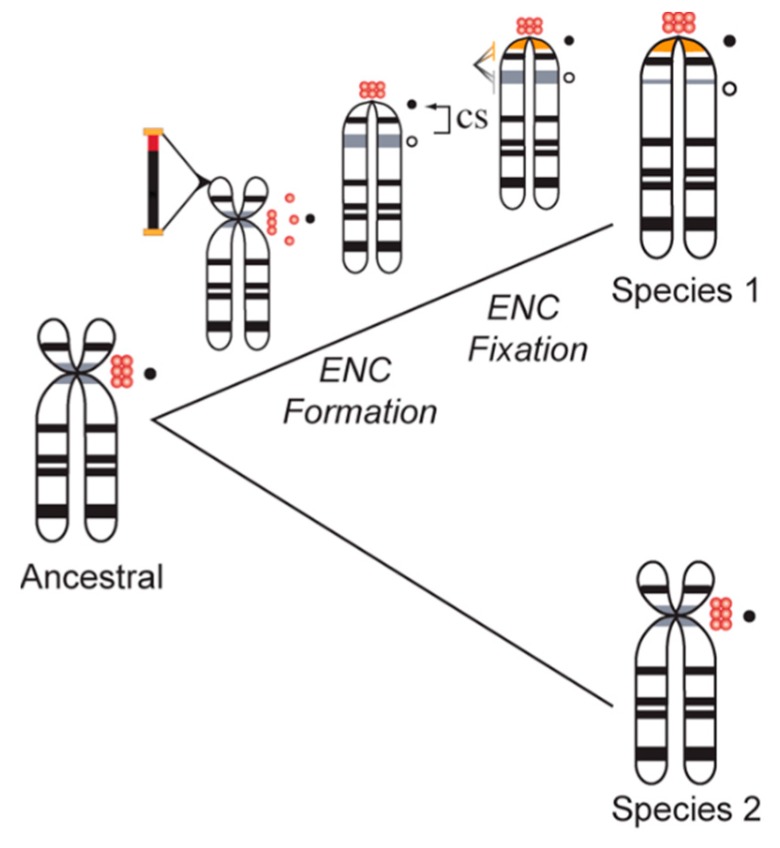
The hypothetical evolution of new centromeres. The ancestral chromosome in this example is submetacentric (the centromere is indicated with red ‘nucleosomes’). The active locus (black dot) carries satellite arrays. Some individual(s) in a population experience the destabilization of the active centromere and formation of a neocentromere, perhaps through the activation of a new TE, resulting in a centric shift (CS). The new centromere is indicated with a black dot, while the latent centromere is indicated with an open circle. The new centromere becomes fixed in a population and eventually gains new satellite arrays (orange), either by interchromosomal seeding from the old centromere (grey) or from the TE itself. Over time, the latent centromere loses its HORs while the new centromere becomes stabilized. In some cases, the ENC can lead to a new species karyotype.

Neocentromeres are not restricted to clinical cases of chromosome instability; shifts in centromere location with no discernable change in intervening gene order distinguish species-specific karyotypes in many eukaryotic taxa. Formerly referred to as centric shifts [102,103], these evolutionary new centromeres (ENCs) [104] (Figure 4) have been characterized in primates, horses, cattle, marsupials, plants, insects, and many other species complexes (see [102,103,104,105,106,107,108] for examples). Moreover, several groups have noted a lack of higher order satellite arrays in newly emerged, functional centromeres, indicating that the formation of homogenized arrays succeeds centromere fixation in a population [109]. It has been proposed that following the fixation of a novel centromere in a species, satellite arrays accumulate to further stabilize the centromere [110,111]. Successive interchromosomal homogenization further support the establishment of large, stable regional centromeres that are rendered species-specific [109,112,113,114,115]. Thus, ENCs accumulate satellite DNA arrays across successive generations as they phylogenetically age, while their immature counterparts lack these types of repetitive sequences (Figure 4). 

Not only has it been established that some recently emerged centromeres lack the higher order satellite arrays characteristic of functional centromeres in a wide variety of organisms, but species in the *Equus* genus carry several centromeres that lack satellite DNA altogether [110,111,116]. Included in those devoid of satellite DNA are ENCs, repositioned to a non-centromeric location following the loss of function at the original centromere [108]. Based on the emerging ENC hypothesis, the recently diverged *Equus* genus, estimated to share a last common ancestor with other genera just 2–3 million years ago despite considerable karyotypic variation, would be predicted to contain de novo centromeres helping drive karyotypic variation that lack satellite DNA. Immuno-FISH experiments using satellite DNA and antibodies against CENP-A completed by Piras et al. [111] identified both functional centromeres lacking satellite DNA as well as satellite repeats present at non-centromeric locations, suggesting the presence of both immature centromeres and ancestral yet inactive centromeric locations, respectively. The identification of a fixed, satellite-free centromere on chromosome 11 in *Equus caballus* presented a distinctive opportunity to test whether there was detectable variability in kinetochore assembly localization on an ENC. ChIP-on-chip analyses in five *Equus* individuals using an antibody against CENP-A revealed at least seven functional centromere epialleles on chromosome 11 dispersed across a region of 500 kb and extending between 80 to 160 kb [117]. The results of these experiments, and recent work in *Equus asinus* [110], demonstrate significant plasticity in CENP-A binding domains among individuals and suggest the potential for centromeres across mammalian species to positionally ‘slide’, resulting in the formation of variable functional epialleles [110,111].

Genome sequencing efforts have further revealed that many eukaryotic species lack centromeres enriched for satellite arrays. For example, sequencing following chromatin immunoprecipitation with antibodies to centromeric proteins CENP-A and CREST, Johnson et al. [118] report a lack of satellite arrays in the centromeres of the recently characterized koala (*Phascolarctos cinereus*) genome, an observation also described in gibbon centromeres and suggestive of the recent evolution of new elements associated with centromere function [119]. Furthermore, this observation has also been documented in a number of other species with small centromeres, ranging from plant species like rice [120] and potato [74] to marsupials like the tammar wallaby [121,122], and fungal species such as *Candida albicans* [123,124]. Taken collectively, new centromere formation is likely not initiated by satellite DNAs; however, satellite DNA is a shared feature of regional centromeres and thus likely promotes their stability. While the introduction of α satellite arrays in human cells can result in the formation of a functional neocentromere, supporting the proposal that satellite DNA is foundational to centromere activity [125,126], the seeding of new ectopic neocentromeres appears to occur in the absence of satellite DNA.

## 4. Satellites and Their Party Friends—Transposable Elements

While satellite DNA is pervasive in the stable, regional centromeres of many species, another class of repetitive element is found within satellite-rich centromeres, ENCs, and neocentromeres: TEs. TEs are repetitive sequences that are able to alter their location in the genome and thus are often considered selfish elements [1,127,128]. Originally characterized by cytogeneticist Barbara McClintock [129], transposable elements can be divided into two categories based on mobility; transposons alter their position directly via a cut and paste mechanism, while retrotransposons move via a copy and paste mechanism through which an RNA intermediate is first created before being reverse transcribed into an identical DNA sequence inserted at a particular genomic locus [130,131]. 

Transposons moving via a cut and paste mechanism, also called type II transposable elements, require a self-encoded enzyme, transposase, in order to move from one locus to another [130,131]. The transposon, flanked by terminal inverted repeats, is recognized by transposase which removes the transposon before reintegrating it at a target location. The gap left behind by transposon excision can be repaired either with, or without, the addition of a replacement transposon. Dissimilarly, retrotransposons, also called type I transposable elements, rely on the transcription of an RNA intermediate as part of their transposition [130,131]. Following transcription, retrotransposon RNA intermediates are reverse transcribed into identical DNA sequences and integrated into a target locus [130,131]. Unlike transposase-mediated mobility, the number of retrotransposons present in a genome increases in number each time they undergo transposition. 

Like satellite DNA, transposable elements form a significant portion of eukaryotic genomes. In fact, due to the ability for many subfamilies to multiply during retrotransposition, TEs can occupy a significant majority of eukaryotic genomes [132,133,134], constituting up to 85% of the maize genome [134] and nearly 50% of the human genome [135]. Historically believed to simply self-propagate, it is now understood that these elements not only comprise a bulk of eukaryotic DNA but also contribute significantly to a wide range of regulatory functions within a genome. Unsilenced TEs have been observed to contain *cis*-regulatory sequences that, due to their motility, have been dispersed broadly throughout the human genome [136,137]. These *cis*-regulatory elements have been shown by several groups to act as promoters, enhancers, and repressors of transcription [138,139,140,141,142]. Using human and mouse cell lines, Sundaram et al. [136] found that 20% of transcription factor binding sites were embedded within transposable elements. Similarly, Cao et al. [142] identified widespread enhancer-like repeats throughout the human genome, many of which were enriched in the mammalian-wide interspersed repeat (MIR) family of short interspersed nuclear elements (SINEs) and the L2 family of long interspersed nuclear elements (LINEs). Moreover, Makarevitch et al. [143] suggest the potential for TEs to provide a mechanism for the upregulation of particular genetic transcripts following abiotic stress in maize via their enhancer-like activity. These studies represent just a fraction of the mounting evidence suggesting that TEs provide necessary regulatory functions within a genome (e.g., see [4,144,145] for reviews).

Despite the high frequency of transposable elements in both human and other eukaryotic genomes, the majority of transposable elements are not actively moving from one genomic locus to another. While mutations have rendered many transposable elements inactive, some have been epigenetically silenced through various mechanisms, such as post transcriptional modifications via RNA interference, DNA and chromatin modifications, and germline silencing. Epigenetic silencing prevents TEs from producing the proteins required for mobility despite a lack of change to the underlying DNA sequence (reviewed in [109,146,147]). 

While satellite DNA is characteristic of centromeres across eukaryotic organisms, the surrounding regions of pericentric heterochromatin are often enriched in TE content. For example, while human centromere cores are enriched for tandem repeat stretches of α satellite DNA, the surrounding heterochromatin regions consist of shorter satellites (e.g., satellites I and II) and primarily two different types of retrotransposons: LINEs and SINEs. Emerging models of centromeric contigs have shown that TE insertions are also found within HOR arrays of the centromere core of all human chromosomes [148,149,150]. This characteristic, coupled with the observations that TEs are often found at both neocentromeres [99,151,152] and ENCs that are devoid of any satellite content [117,118,153,154], suggests a potential role for TEs in centromere function independent of resident satellite DNA.

While the exact role TEs play in centromere function is not currently known, several features of centromeric TEs have been revealed. For example, epigenetic silencing of transposable elements appears to be critical in maintaining proper centromere function and chromosome segregation [147,155,156]. In mice, activating regularly silenced long terminal repeat (LTR) and non-LTR retrotransposons at centromeric regions has led to defects in both meiosis and chromosomal segregation, suggesting the necessity of epigenetically silent transposable elements for appropriate centromere function [156]. Undermethylation of centromeric retroelements in interspecific hybrids led to centromere destabilization and chromosome instability [157,158], indicating that tight regulation of TE activity underlies centromere stability. Moreover, studies have suggested a link between centromeric retrotransposons and the silencing of satellite DNA in the centromere, as well as a link between satellite DNA and the silencing of retrotransposons. May et al. [159] describe this relationship in *Arabidopsis thaliana,* in which satellite-derived transcripts are epigenetically silenced in part due to the insertion of transposable elements. Phylogenetic analyses and TE annotations have led to the observation that species-specific [118] and recently active [160] or hot TEs [152,161] are often the type of element found within centromere cores, while divergent and ancestral TEs are relegated to the hypermutated satellites [30,40,149] found in the outer strata of the centromere and pericentromere (Figure 1a,b). 

It has been suggested that a close evolutionary relationship exists between centromeric TEs and the birth of new satellite families (Figure 3a). In the plant species *Aegilops speltoides*, a 250 base pair repeat satellite array family is present at centromeres [162]. While not identical to that of a transposable element, this satellite DNA sequence shares high similarity to portions of a transposable element: Ty3/gypsy-like retrotransposons. Furthermore, this phenomenon has been observed in other model species as well, including members of the *Arabidopsis* [163], *Drosophila* [164], and *Cetacean* [165] genera. A recent study observed that tandem dimers of TEs form during bursts of TE activity and may serve as fodder for the evolution of satellite arrays, as was found for the *hobo* element in Drosophila [166]. It has been proposed that large-scale mutations, insertions and deletions within centromeric TEs followed by unequal crossing over or even seeding across chromosomes, may give rise to novel tandem repetitive elements found highly enriched at centromeres [112,167,168,169,170]. These processes are thought to act as part of the host-defense mechanisms to inactivate mobile elements ([171,172] but see [173]) or prevent non-allelic homologous recombination ([174,175] and reviewed in [109,176,177]). 

Within plants, allopolyploidy presents a unique opportunity for the evolution of centromeric sequences from resident TEs. Following allopolyploidization, and during the genomic instability that ensues, centromeric TEs from the different progenitor genomes may become activated [178]. Evidence has been found in *Gossypium* (cottons) that such activation likely occurred, resulting in the integration of TEs from one genome into another, and subsequent proliferation among centromeres [179]. This activity, coupled with the exposure of new genomic material in the polyploid state, provides an opportunity for competition among multiple, newly emerged centromere repeats and the possible selection for repeats that are more conducive to supporting centromere nucleosome structure [180].

## 5. Transcription in the Centromere—Let’s Get the Party Started!

While a function for satellite DNAs in kinetochore assembly and/or stability has been inferred since their discovery (e.g., [181,182,183]), a common misconception has been that these sequences were not actively transcribed into RNAs (but see [184] and references therein from the 1960s). Undoubtedly, the discovery that satellite DNAs are transcriptionally viable has led to a shift in how we view centromeric chromatin [185,186]. Soon after the discovery of satellite DNA in cesium gradients, electron microscopy revealed RNA at plant and animal centromeres [187,188], although satellites themselves were not directly linked to active transcription. Furthermore, examination of the linear organization of histones within centromeres using chromatin fiber FISH revealed that CENP-A nucleosome domains were interrupted by nucleosomes containing H3K4me2 [189,190] and H3K36me2 [191], epigenetic marks of active transcription. Using chromatin immunoprecipitation, Choi et al. reported the detection of RNA polymerase II at centromeres in the fission yeast *Schizosaccharomyces pombe* [192]. Further analyses have identified the presence of RNA polymerase II at centromeres in humans [193], flies [194,195], and budding yeast [196], among others. The presence of RNA polymerase II at these sites suggested active transcription occurring from the DNA present at centromeres: satellites and TEs. 

Transcripts originating from centromeric satellite DNA and TEs have now been observed in a variety of species across eukaryotes [122,159,186,194,196,197,198,199,200,201,202,203,204], while some species, such as *S. pombe,* exhibit transcription of boundary elements (e.g., tRNAs [205]). Thus, centromeric RNAs are a conserved component of the centromere, despite a lack of sequence conservation across these regions. Recent work implicates centromere transcription as integral to centromere function, impacting the pivotal event in centromere assembly: the loading of newly synthesized CENP-A histones. For example, in budding yeast [196] and human artificial chromosomes [191,206,207], transcriptional silencing of centromeric DNA has been shown to lead to a failure to maintain proper centromere function. In human artificial chromosomes, this malfunction was attributed to the inability to load new CENP-A during mitosis to G1. Conversely, upregulation of satellite transcripts is also detrimental to centromere function, causing the removal of the CENP-A histone variant [196,200,207,208] as well as cellular instability [196,200,209,210,211,212,213]. Intriguingly, several proteins involved in the kinetochore assembly cascade are either RNA binding proteins or have been demonstrated to associate with RNAs in a complex, including CENP-A [199], CENP-C [202,214], and KNL2/M18BP1 [215]. While the transcriptional framework underlying centromere assembly is not fully understood (but see [109,186]), several mechanisms have been proposed that can promote transcription within regional centromeres. Early work in plants [199] and marsupials [122] supported the hypothesis that centromeric TEs promote transcription, and their ability to transcribe neighboring satellites is implied by the presence of bi-directional promoters within these TEs [200,216,217,218]. More recently, it has been hypothesized that non-B form DNA facilitated by dyad symmetries and CENP-B binding within centromeres may facilitate transcription [219]. While there is a clear connection between transcription and centromere nucleosome assembly [194,195,199,200,220,221], how and when this occurs during the cell cycle remains elusive. 

Transcription has also been linked to the emergence of new centromeres. In a human neocentromere case, a L1 was found transcribed and actively demarcating the CENP-A domain of the new centromere [152,222]. Given earlier work demonstrating that demethylation of centromeric TEs led to increased activity [158], release of ectopic TEs from a silenced state may facilitate their transcription and subsequent recruitment of CENP-A nucleosomes, leading to the rescue of acentric chromosome fragments following the inactivation of the native centromere. How an ectopic site becomes activated, enabling the recruitment of CENP-A nucleosomes in the absence of chromosome damage, as is implied by centromere repositioning events, is unknown. If multiple inversion events, insertion events by active TEs, or simply deletions of part of an HOR array lead to the interruption of the native satellite array, destabilization of the kinetochore assembly cascade may follow, necessitating a rescue centromere elsewhere on the chromosome. Perhaps the most recent TE insertions in a genome allow ectopic centromere formation as such elements have yet to experience silencing by host defense mechanisms. Under this model, some mechanism must prevent the activation of ectopic centromeres at these hot elements when native centromeres are still functional to prevent the formation of dicentric chromosomes and subsequent breakage-fusion-bridge cycles [223,224,225]. 

## 6. Conclusions

The influence of repeated DNAs on eukaryotic genomes is often presented in the framework of the logical fallacy that repeated DNA should no longer be considered inconsequential ‘junk DNA’. Contextualizing repeated DNAs under such as false descriptor, even when presented as an oft challenged and subsequently defeated cliché, undercuts not only the long-standing validity of studying repeated DNAs, but the growing impact the field of repeat DNA biology has had on our understanding of eukaryotic genome biology and evolution. The repeats found at centromeres are an excellent case in point. There is little doubt that centromeric repeats, including both satellites and TEs, are integral to centromere function and stability as well as the evolution of novel karyotypes. The models discussed herein are not all-inclusive yet demonstrate the unique processes that have allowed for significant species-specific variation among repetitive DNAs despite a simultaneously foundational role in genome stability and regulation. As we gain an understanding of the evolutionary forces that influence the constitution of centromeric DNA, we can start to unravel the impact centromeric sequences have on both maintaining chromosome stability within a species and karyotypic change during species evolution.
